# American Ginseng Regulates Gene Expression to Protect against Premature Ovarian Failure in Rats

**DOI:** 10.1155/2015/767124

**Published:** 2015-02-01

**Authors:** Lei Zhu, Ji Li, Nannan Xing, Dongwei Han, Haixue Kuang, Pengling Ge

**Affiliations:** ^1^Department of Pharmacology, School of Basic Medical Sciences, Heilongjiang University of Chinese Medicine, 24 Heping Road, Harbin 150040, China; ^2^The Key Laboratory of State Administration of Traditional Chinese Medicine of the People's Republic of China, Department of Formulas of Traditional Chinese Medicine, School of Basic Medical Sciences, Heilongjiang University of Chinese Medicine, Harbin 150040, China; ^3^The Key Laboratory of Chinese Ministry of Education, Department of Traditional Chinese Medicine, School of Pharmacology, Heilongjiang University of Chinese Medicine, Harbin 150040, China; ^4^The Key Laboratory of Myocardial Ischemia (Harbin Medical University) of Chinese Ministry of Education, Harbin 150086, China

## Abstract

Premature ovarian failure (POF) is defined as lost ovarian functions before the age of 40. Three possible molecular markers (*PLA2G4A*, * miR-29a*, and *miR-144*) have been identified in our previous study by integrated analysis of mRNA and miRNA expression profiles. The present study aimed to evaluate American ginseng root's protective potential against POF by studying transcriptional and protein variations between American ginseng treatments and controls in rats. 4-Vinylcyclohexene diepoxide (VCD) was administered to rats for 14 days to induce POF. Additionally, American ginseng was administered to POF rats for one month, and *PLA2G4A*, *miR-29a*, and *miR-144* expressions were measured in rat ovaries by qRT-PCR. PLA2G4A protein expression was examined by Western Blot, and PGE_2_, LH, FSH, and E2 serum levels were detected by ELISA. *PLA2G4A* mRNA and protein were downregulated in American ginseng-treated rats, *miR-29a* and *miR-144* levels increased, and PGE_2_ serum levels decreased, while LH, FSH, and E2 increased compared to POF induction alone. Analysis of transcriptional and protein variations suggested that American ginseng protects the ovary against POF by regulating prostaglandin biosynthesis, ovulation, and preventing ovarian aging. High hormone levels (PGE_2_, FSH, and LH) were reduced, and E2 secretion approached normal levels, leading to improved POF symptoms and abnormal ovulation.

## 1. Introduction

Premature ovarian failure (POF), also known as premature ovarian insufficiency, is characterized by amenorrhea for at least four to six months before the age of 40 with sex steroid deficiency and raised serum concentrations of follicle-stimulating hormone (FSH) of more than 40 IU/L occurring at least 1 month apart [1]. Clinical diagnosis of POF is recognized as amenorrhea (>6 months) with estrogen deficiency and high concentrations of luteinizing hormone (LH) and FSH (>20 IU/L) before the age of 40 [2]. Ovarian insufficiency often starts as secondary amenorrhea with increased FSH levels, which is also known as transitional ovarian failure [3]. Many risk factors may contribute to POF, such as physical and chemical factors, radiation and chemotherapy, ovarian failure following hysterectomy, autoimmune diseases, or hereditary factors. There is still no effective clinical treatment for POF because its etiology still remains unclear. Many women with POF are advised to undergo long-term hormone replace therapy (HRT), which helps to relieve the symptoms of perimenopausal syndrome that can have significant impact on the woman's quality of life. In the current study, we successfully induced the occurrence of POF in rats using an ovotoxic chemical 4-vinylcyclohexene diepoxide (VCD), which can specifically accelerate the atresia of primordial and primary follicles in rodents when primordial follicles are depleted [4].

The herb American ginseng (*Panax quinquefolius* L., Araliaceae) is one of the top ten selling natural health products in the United States [5]. American ginseng is used as an antifatigue drug and as an immunostimulant during periods of stress. In a study by Duda et al., American ginseng was shown to induce the expression of pS2, a protein that may exhibit estrogen-like effects on estrogen receptor-positive breast cancer cells [6]. In addition, American ginseng was previously studied in the mouse adipose cell line 3T3-L1 to determine its potential to inhibit proliferation, decrease the percentage of cells in S phase, and induce the expression of adiponectin, a euglycemic agent [7]. Another study showed that ginseng saponin treatment can ameliorate central nervous system (CNS) disorders and neurodegenerative diseases [8, 9]. In addition, several studies have indicated that American ginseng saponins can significantly improve cognitive abilities and emotional fluctuations [10–12].

Ginseng has been used as a nutritional supplement in East Asia for thousands of years and has recently gained popularity in the West because of its various pharmacological properties. Many experimental studies have shown that ginseng has estrogenic [6], anticancer [13], and hypoglycemic effects [14, 15] and can improve impaired memory and learning [16], which may contribute to its effect on the prevention or treatment of POF secondary diseases, such as dementia, diabetes mellitus, metabolic syndrome, osteoporosis, and certain cancers. Thus, we hypothesized that American ginseng may exert protective effects against POF and its associated complications.

In the last few years, microRNAs (miRNAs) have been found as new cell regulators for messenger RNA (mRNA) gene expression [17, 18]. miRNAs are small, noncoding RNAs in length of 20–24 nucleotide that can repress mRNA expression by binding to the 3′untranslated regions (UTR) of target mRNA, which leads to translational repression, mRNA cleavage, and deadenylation [19, 20]. Each miRNA can influence the expression of multiple target mRNAs and each mRNA can be regulated by several miRNAs [21].

In this preliminary observational study, we confirmed that* PLA2G4A* was overexpressed in POF ovarian tissues. We hypothesized that* PLA2G4A* was a candidate gene indirectly involved in POF occurrence and development by increasing prostaglandin concentration, which is important in ovulation. We also determined efficient associations between expression of both miRNAs and mRNA in target gene sets, by investigating the miRBase, MiRanda, and miRDB databases [22]. The expression of* miR-144* and* miR-29a* has been shown to suppress* PLA2G4A *transcription or translation or to cut the mRNA so that it is targeted for degradation. In our previous study, we also demonstrated that* miR-29a* and* miR-144 *expression decreased in POF ovarian tissues. Therefore, the mechanism by which American ginseng is protective against POF may involve regulation of such potential targeted gene and miRNAs.

*PLA2G4A* expression can induce arachidonic acid (AA) release [23]. Prostaglandin (PG) biosynthesis is dependent on AA release [24] and synthetases, including PG endoperoxide synthase (PTGS) and specific PG synthase enzymes [25]. AA is converted to PGG2 through the bifunctional enzymes cyclooxygenase- (COX-) 1 or COX-2, resulting in the intermediate PGH_2_, which is ultimately converted into prostaglandin estradiol (PGE_2_) and other subtypes or thromboxanes (TX) by cell-specific synthases [26]. Matsumoto and Espey previously showed that prostaglandins regulate a series of important physiological processes, including female fertility and the reproductive lifespan [27, 28]. During the 1970s, the importance of prostaglandins and their connection to ovulation became increasingly apparent. PGE_2_ is secreted by follicles and regulates ovulation [25, 29].* PLA2G4A* expression is important in this process, as injection of rats with the specific* PLA2G4A* inhibitor arachidonyl trifluoromethyl ketone significantly decreased intraovarian bursal ovulation and total ovarian PGE_2_ synthesis [30]. These data highlight a role for* PLA2G4A* activity in ovulation and PGE_2_ synthesis.

Based on previous studies and the various pharmacological properties of American ginseng, our study aimed to establish the association between American ginseng and POF. Furthermore, we explored the molecular mechanism by which American ginseng may regulate gene expression and hormone secretion to protect against POF.

## 2. Materials and Methods

### 2.1. Animals and Treatment

Adult female Sprague Dawley rats (200 ± 20 g weight) were provided by the Yisi Laboratory Animal Technology Co., Ltd. (Changchun, China).

Rats were individually housed under constant temperature (20 ± 1°C), humidity (50 ± 5%), and light (12 h/d) conditions with standard pellet diet and water provided* ad libitum*. The animal protocol was approved by the Animal Experimental Ethical Committee of Heilongjiang University of Chinese Medicine.

Fifteen female Sprague Dawley rats (3 months old) were divided into three groups: control group (*n* = 5), 4-vinylcyclohexene diepoxide- (VCD-) induced (model) group (*n* = 5), and American ginseng-treated group (*n* = 5). VCD-induced rats and American ginseng-treated rats were both intraperitoneally (i.p.) injected with sesame oil or sesame oil plus VCD (80 mg/kg/day) for 14 consecutive days. At the same time, American ginseng-treated rats were administered American ginseng (2.25 g/kg body weight) by daily oral gavage. The control group was given equal volumes of physiological saline. After the final VCD injection, the American ginseng-treated group continued to drench for 30 days. Animals had daily access to food and water* ad libitum* for observation of toxicity and mortality.

The dosage, route of administration, and duration of treatment were based on our previous study. After 30 days, ovaries and blood serum were collected, weighed, rapidly frozen by liquid nitrogen, and stored at −80°C. The ovary weight index by the following formula: ovary weight index = ovarian weight/body weight.

### 2.2. RNA Extraction and qRT-PCR

Following the manufacturer's instructions, total RNA was extracted from whole ovaries by TRIzol (Invitrogen, Carlsbad, CA, USA) and an miRNA easy minikit (Qiagen, Valencia, CA, USA). RNA concentrations were determined with a NanoDrop ND-1000 spectrophotometer (Wilmington, DE, USA). Finally, RNA integrity was assessed by denatured agarose gel electrophoresis.

For qRT-PCR experiments, total isolated RNA was reverse-transcribed into cDNA with an AccuPower RocketScript RT Premix (Bioneer, Daejeon, Korea) according to the manufacturer's instructions. The experiment was performed in duplicate using AccuPower GreenStar qPCR master mix according to the manufacturer's instructions. Tables 1 and 2 show the primer sequences used to amplify fragments. The data were normalized to expression levels of the housekeeping genes *β*-actin and U6, respectively, and equation 2^−ΔΔC(t)^ was used to calculate relative expression levels. PCR products were analyzed by agarose gel electrophoresis to ensure that only a single band of the expected size was amplified.

### 2.3. Enzyme-Linked Immunosorbent Assay (ELISA) Analysis

PGE_2_, FSH, LH, and E2 serum levels were assayed with an ELISA kit (R&D Quantikine, R&D Systems Inc., Minneapolis, MN, USA). Each test was performed in triplicate, and five independent experimental results were used for statistical analysis.

### 2.4. Western Blot Analysis

PLA2G4A protein expression was verified in ovarian tissue samples by Western blot, and *β*-actin was used as a loading control.

Protein aliquots (50 *μ*g) from ovaries treated with VCD, VCD and ginseng or with no treatment was loaded onto a 12% sodium dodecyl sulfate gel, and electrotransferred to a PVDF membrane. Membranes were blocked in 10% nonfat dried milk. The membrane was incubated with rat anticytosolic phospholipase A2 (PLA2G4A; Santa Cruz Biotechnology, Santa Cruz, CA, USA, 1 : 1000) at 4°C for 12 h. Blots were incubated with HRP-conjugated IgG (1 : 1000; Sigma-Aldrich, St. Louis, MO, USA) at room temperature for 1 h. The PLA2G4A protein was detected by chemiluminescence (Pierce, Rockford, IL, USA).

### 2.5. Statistical Analysis

The data were expressed as mean ± SEM, and the means among different groups were analyzed by one-way ANOVA post hoc tests. *P* < 0.05 was considered statistically significant.

## 3. Results

### 3.1. Differential Expression Analysis of mRNA, miRNAs, and Protein in POF

**Figure 1:**
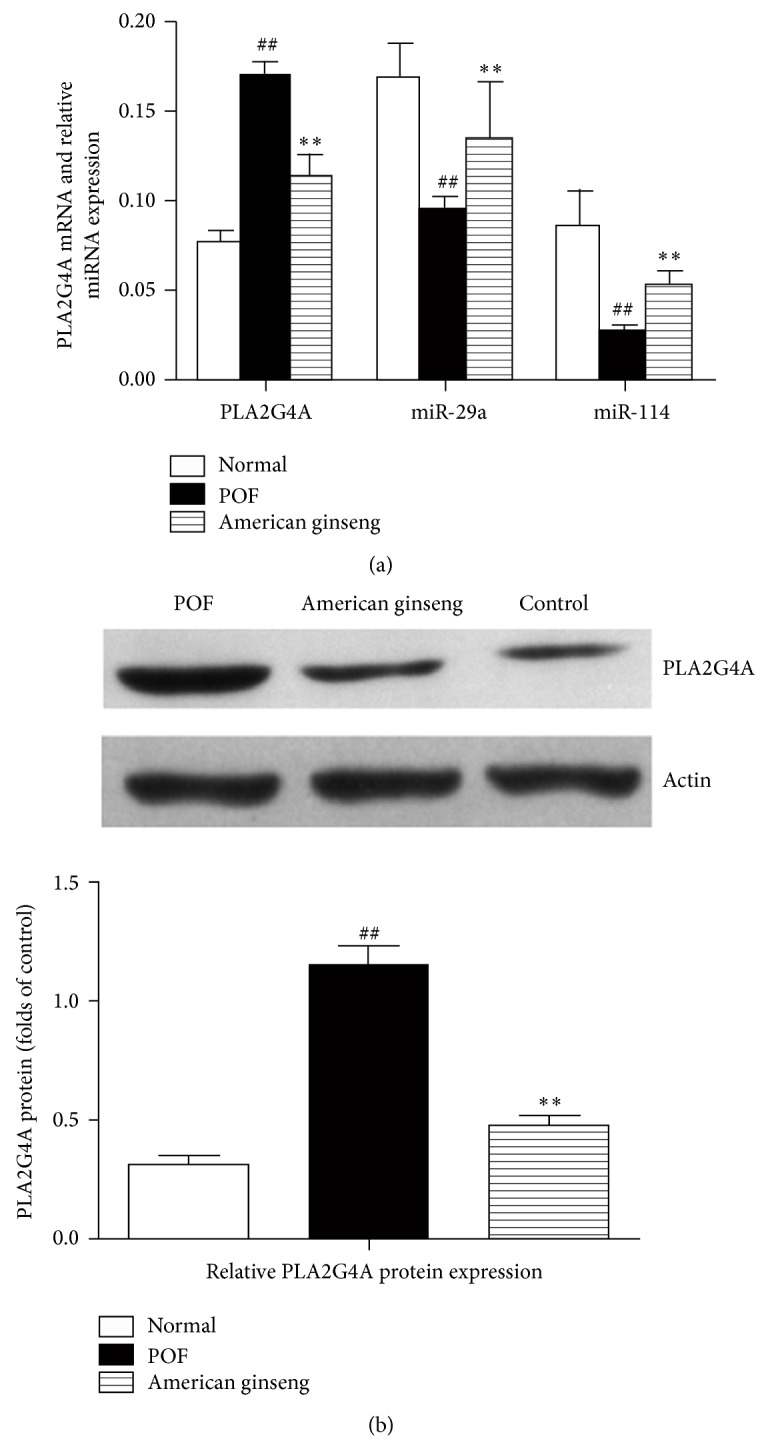
Validation of differential* miR-29a*,* miR-144*,* PLA2G4A mRNA, *and PLA2G4A protein expression in American ginseng-treated POF tissues. (a) miR-29a and miR-144 and* PLA2G4A *levels in ginseng-treated ovaries were determined by qRT-PCR. (b) Western blotting for PLA2G4A protein in the absence or presence of American ginseng. The expression level trended toward control levels. Data were expressed as mean ± SEM from five independent experiments performed in duplicate. ^**^ *P* < 0.01 versus VCD-treated groups and ^##^ *P* < 0.01 versus control.

To determine whether* PLA2G4A* expression changed upon American ginseng treatment, we performed RT-PCR and found that* PLA2G4A* expression significantly decreased after American ginseng treatment. Additionally, the upstream miRNAs* miR-29a* and* miR-144* were downregulated compared to the POF model (*P* < 0.05, Figure 1(a)). Western blot analysis confirmed that PLA2G4A protein expression was downregulated compared to POF ovarian tissue (Figure 1(b)).

### 3.2. Ovary Weight Index

**Figure 2:**
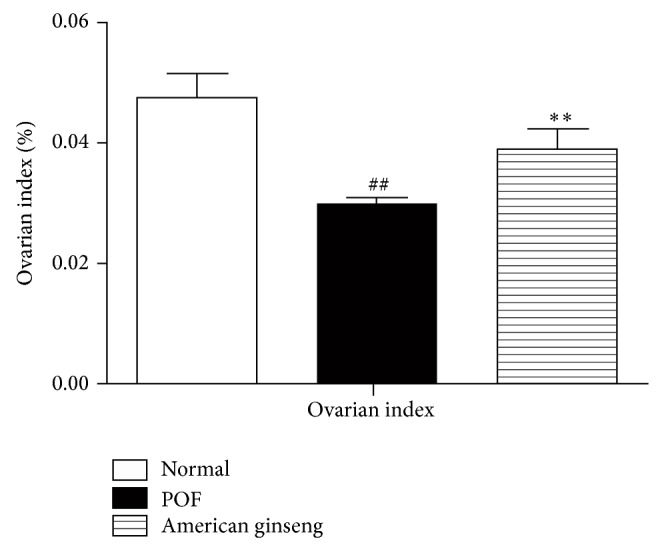
Ovary weight. Ovarian index = ovarian weight/body weight; data were expressed as mean ± SEM. ^**^ *P* < 0.01 versus VCD-treated groups and ^##^ *P* < 0.01 versus control.

Compared to the control group, POF ovaries had clearly atrophied. Ovaries from American ginseng-treated rats had also mildly atrophied but not to the extent of POF ovaries (Figure 2).

### 3.3. American Ginseng Treatment Partially Rescues Serum Hormones Levels

**Figure 3:**
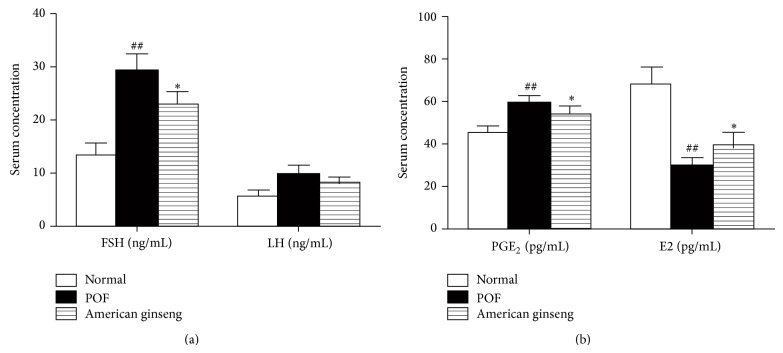
PGE_2_, E2, FSH, and LH serum levels. PGE_2_, FSH, LH, and E2 serum levels were detected by ELISA. Data are summarized and presented as mean ± SEM from four independent experiments. ^*^ *P* < 0.05 versus VCD-treated groups and ^##^ *P* < 0.01 versus control.

By ELISA, we determined that FSH and LH serum levels increased after POF induction and were partially rescued by American ginseng treatment (Figure 3(a)). However, values of LH serum level were differentially expressed but showed no significant fold change (*P* > 0.05) between the treatments. Similarly, PGE_2_ serum levels increased in POF animals compared to controls. Serum levels were partially rescued by American ginseng treatment. In contrast, POF induction decreased E2 levels, which were partially restored by American ginseng treatment, bringing them closer to control levels (Figure 3(b)).

## 4. Discussion

POF may be caused by several risk factors, including chemicals, iatrogenic agents, ovarian failure following hysterectomy, autoimmune diseases, or hereditary factors. However, the underlying molecular etiology remains complex. In our early studies, we integrated the data of several differentially expressed miRNAs and related mRNAs in ovarian tissue from POF rat models to identify potential targeted genes and estimated their efficient associations with POF [22]. Therefore, the potential targeted genes we identified may be those candidate genes regulated by American ginseng. In this study, we used the same treatment regimens and found that American ginseng significantly reduced* PLA2G4A* expression and increased* miR-144* and* miR-29a* expression in POF ovarian tissues compared to POF induction alone. We found that the differentially expressed genes were involved in prostaglandin biosynthesis, which plays an important role in ovulation. As expected, PGE_2_ concentrations decreased after American ginseng treatment compared to POF induction alone. Furthermore, the increased ovary weight index and E2 concentrations in American ginseng-treated rats compared to POF induction alone suggest that American ginseng partially reduces ovarian atrophy and prevents abnormal ovulation and subsequent occurrence and development of POF. These data further suggest that American ginseng has potent protective effects against VCD-induced POF. In the current study, we detected mRNA and miRNAs that are regulated by American ginseng and are involved in important biological events likely contributing to POF development, such as prostaglandin biosynthesis, ovulation, hormone secretion, and ovarian aging.

A series of clinical findings have suggested that* PLA2G4A* is indirectly involved in ovulation [25]. When* PLA2G4A* was knocked out in mouse ovaries, ovulation and fertilization rates were significantly reduced compared to control littermates [31]. Previous studies have shown that ovulation is preceded by the induction of* PLA2G4A* expression in the granulosa cells of bovine ovulatory follicles [25]. In our previous studies, we confirmed that* PLA2G4A *was overexpressed in POF ovaries and hypothesized that upregulation of* PLA2G4A* may indirectly result in the occurrence and development of POF by increasing prostagl and in concentration.

PLA2G4A is a catalytic enzyme in AA biosynthesis [29]. Prostaglandin biosynthesis requires AA release and* PLA2G4A* is essential for proper induction of PTGS2, an isoform of the PTGS synthetase in prostaglandin biosynthesis [32]. PTGS2 is necessary for the response to proinflammatory stimuli [33]. Kurusu et al. have shown that PLA2G4A cellular and temporal expression coincides with PTGS2 expression in follicles [31]. The LH surge induces ovarian PTGS2 expression and then induces an increase in PGE_2_ (a subtype of prostaglandin) production in preovulatory follicles, which are two critical factors in oocyte maturation [34] and ovulation [27]. Previous studies have also demonstrated that* PLA2G4A* is required for prostaglandin biosynthesis. In the current study, our data suggest that American ginseng can suppress* PLA2G4A* mRNA and PLA2G4A protein expression in ovarian tissues. Furthermore, American ginseng treatment reduced LH secretion, though not significantly, in serum of POF rats, which may lead to the downregulation of PGE_2_ and the rescue of abnormal ovulation.

It has previously been reported that prostaglandin is important for ovulation. PLA2G4A contributes to AA release, a substrate necessary for the release of proinflammatory mediators, such as PGs and leukotrienes [35]. Our data revealed that PGE_2_ serum levels decreased in POF rats treated with American ginseng. At present, ovulation is primarily considered an acute and self-steering inflammatory reaction, including local edema, white blood cell release from the blood vessel, and activation of proteolytic enzymes and collagenolytic enzymes [36]. These changes result in follicle wall rupture and mature egg release. Recently,* PLA2G4A* mRNA levels were found to increase during the proresolving phase of the acute inflammatory process [37]. PGE_2_ can induce inflammation, fever, and pain and induce ovulation. Animal studies have shown that the number of CD68+ cells, macrophages, and neutrophils significantly increased in the follicular fluid after treatment with PGE_2_, which can activate the expression of ovarian proteolytic enzymes and further promote follicular rupture and egg release [38]. Ristimäki et al. injected PGE_2_ or its derivatives into monkeys treated with indomethacin, an inflammation inhibitor, prior to ovulation to restore ovarian function [39]. Therefore, increasing PGE_2_ levels may indirectly lead to POF. Regulation of PGE_2_ levels may be American ginseng's primary mechanism of action to protect against POF.

In addition, PGE_2_ affects neuroendocrine cells in the hypothalamus, which can induce gonadotropic releasing hormone (GnRH) release. GnRH stimulates the anterior pituitary gland to secrete LH and FSH [2]. LH can regulate hormone production, and FSH is responsible for follicle development. In this study, we found that ovary weight ratios increased after American ginseng treatment compared to POF induction alone, and their ratios tended to decrease towards the control rats. The restoration of ovarian weight may contribute to restoring ovarian function. Additionally, we observed increased serum levels of E2 after treatment with American ginseng and decreased FSH and LH secretion by the hypothalamic-pituitary-ovarian axis. Therefore, the decrease in FSH and LH levels may be due to the reduction of PGE_2_ and increase in E2 induced by American ginseng in POF rats. The recovery of hormone production further prevented the primordial follicle pool from being abnormally depleted.

To further confirm whether the differential expression of* PLA2G4A* was directly induced by American ginseng, we performed qRT-PCR analysis to determine whether American ginseng affected the expression of* miR-144* and* miR-29a*, both of which can suppress* PLA2G4A* expression. Our results suggested that* PLA2G4A* downregulation might be secondary to the upregulation of* miR-144* and* miR-29a* induced by American ginseng treatment. However, it is also possible that American ginseng may regulate* miR-144* and* miR-29a* expression to specifically restore* PLA2G4A* levels. Indeed, Yao et al. previously showed that* miR-29a* expression was significantly suppressed in FSH-treated cultured rat granulosa cells [40]. Moreover, a recent study showed that follicular thyroid carcinoma tissues had decreased* miR-144* expression, resulting in activated mTOR signaling [41], which can accelerate ovarian aging and induce POF [42]. In our previous study, we demonstrated that* miR-29a* and* miR-144* expression decreased in POF ovarian tissues. This inverse correlation might verify our current results, indicating that upregulated* miR-29a* and* miR-144* induced by American ginseng treatment may play a significant role in protecting ovarian function by regulating the response to hormone stimulation and preventing ovarian aging.

From our analysis, we found that American ginseng protects against POF by altering mRNA and miRNAs expression and hormone levels. The changes in the* PLA2G4A* and* miR-29a* and* miR-144* genes suggest that American ginseng exerts its effect by regulating prostaglandin biosynthesis, ovulation, and preventing ovarian aging. The high hormone levels (PGE_2_, FSH, and LH) were reduced and E2 secretion approached normal levels upon American ginseng treatment, which led to improved symptoms of POF and abnormal ovulation. Although further research is necessary to elucidate the active constituents of American ginseng and to validate the function of ginseng in POF women and rats, our study expands our understanding of American ginseng's pharmacological activities as an anti-POF agent.

## Figures and Tables

**Table 1:** qRT-PCR primers for mRNA.

mRNA	Forward primer sequence [5′→3′]	Reverse primer sequence [5′→3′]
Pla2G4a	GACGCAGCGGTAGCAGAT	TCAAGG GATACGGCAGGT
*β*-actin	GTCAGGTCATCACTATCGGCAAT	AGAGGTCTTTACGGATGTCAACGT

**Table 2:** qRT-PCR primers for miRNA.

miRNA	DNA sequences
rno-miR-29a-3p	5′-CGTAGCACCATCTGAAATCGGTTA-3′
rno-miR-144-5p	5′-CGCGGGATATCATCATATACTGTAAGT-3′
U6	5′-ACACGCAAATTCGTGAAGCGTTCC-3′

## References

[1] de Vos M., Devroey P., Fauser B. C. (2010). Primary ovarian insufficiency. *The Lancet*.

[2] Dixit H., Rao L., Padmalatha V. (2010). Genes governing premature ovarian failure. *Reproductive BioMedicine Online*.

[3] Knauff E. A. H., Eijkemans M. J. C., Lambalk C. B. (2009). Anti-Müllerian hormone, inhibin B, and antral follicle count in young women with ovarian failure. *The Journal of Clinical Endocrinology & Metabolism*.

[4] Hoyer P. B., Sipes I. G. (2007). Development of an animal model for ovotoxicity using 4-vinylcyclohexene: a case study. *Birth Defects Research B*.

[5] Wang C. Z., Mehendale S. R., Yuan C. S. (2007). Commonly used antioxidant botanicals: active constituents and their potential role in cardiovascular illness. *The American Journal of Chinese Medicine*.

[6] Duda R. B., Taback B., Kessel B. (1996). pS2 expression induced by American ginseng in MCF-7 breast cancer cells. *Annals of Surgical Oncology*.

[7] Yeo C.-R., Lee S.-M., Popovich D. G. (2011). Ginseng (*Panax quinquefolius*) reduces cell growth, lipid acquisition and increases adiponectin expression in 3T3-L1 cells. *Evidence-Based Complementary and Alternative Medicine*.

[8] Christensen L. P. (2008). Chapter 1 ginsenosides: chemistry, biosynthesis, analysis, and potential health effects. *Advances in Food and Nutrition Research*.

[9] Rausch W.-D., Liu S., Gille G., Radad K. (2006). Neuroprotective effects of ginsenosides. *Acta Neurobiologiae Experimentalis*.

[10] Hai Y. B., Zhang J., Soo J. Y. (2005). Memory enhancing and neuroprotective effects of selected ginsenosides. *Archives of Pharmacal Research*.

[11] Wang Y.-Z., Chen J., Chu S.-F. (2009). Improvement of memory in mice and increase of hippocampal excitability in rats by ginsenoside Rg1's metabolites ginsenoside Rh1 and protopanaxatriol. *Journal of Pharmacological Sciences*.

[12] Zhang G., Liu A., Zhou Y., San X., Jin T., Jin Y. (2007). Panax ginseng ginsenoside-Rg_2_ protects memory impairment via anti-apoptosis in a rat model with vascular dementia. *Journal of Ethnopharmacology*.

[13] Luo X., Wang C.-Z., Chen J. (2008). Characterization of gene expression regulated by American ginseng and ginsenoside Rg3 in human colorectal cancer cells. *International Journal of Oncology*.

[14] Vuksan V., Sievenpiper J. L., Wong J. (2001). American ginseng (*Panax quinquefolius* L.) attenuates postprandial glycemia in a time-dependent but not dose-dependent manner in healthy individuals. *The American Journal of Clinical Nutrition*.

[15] Vuksan V., Sievenpiper J. L., Koo V. Y. Y. (2000). American ginseng (*Panax quinquefolius* L) reduces postprandial glycemia in nondiabetic subjects and subjects with type 2 diabetes mellitus. *Archives of Internal Medicine*.

[16] Li Z., Guo Y. Y., Wu C. F., Li X., Wang J. H. (1999). Protective effects of pseudoginsenoside-F_11_ on scopolamine-induced memory impairment in mice and rats. *Journal of Pharmacy and Pharmacology*.

[17] He L., Hannon G. J. (2004). MicroRNAs: small RNAs with a big role in gene regulation. *Nature Reviews Genetics*.

[18] Calin G. A., Croce C. M. (2006). MicroRNA signatures in human cancers. *Nature Reviews Cancer*.

[19] Bartel D. P. (2004). MicroRNAs: genomics, biogenesis, mechanism, and function. *Cell*.

[20] Bentwich I., Avniel A., Karov Y. (2005). Identification of hundreds of conserved and nonconserved human microRNAs. *Nature Genetics*.

[21] Chiang H. R., Schoenfeld L. W., Ruby J. G. (2010). Mammalian microRNAs: experimental evaluation of novel and previously annotated genes. *Genes & Development*.

[22] Kuang H., Han D., Xie J., Yan Y., Li J., Ge P. (2014). Profiling of differentially expressed microRNAs in premature ovarian failure in an animal model. *Gynecological Endocrinology*.

[23] Monget P., Bondy C. (2000). Importance of the IGF system in early folliculogenesis. *Molecular and Cellular Endocrinology*.

[24] Joyce I. M., Pendola F. L., O'Brien M., Eppig J. J. (2001). Regulation of prostaglandin-endoperoxide synthase 2 messenger ribonucleic acid expression in mouse granulosa cells during ovulation. *Endocrinology*.

[25] Diouf M. N., Sayasith K., Lefebvre R., Silversides D. W., Sirois J., Lussier J. G. (2006). Expression of phospholipase A2 group IVA (PLA2G4A) is upregulated by human chorionic gonadotropin in bovine granulosa cells of ovulatory follicles. *Biology of Reproduction*.

[26] Crofford L. J. (2001). Prostaglandin biology. *Gastroenterology Clinics of North America*.

[27] Matsumoto H., Ma W., Smalley W., Trzaskos J., Breyer R. M., Dey S. K. (2001). Diversification of cyclooxygenase-2-derived prostaglandins in ovulation and implantation. *Biology of Reproduction*.

[28] Espey L. L. (2006). Comprehensive analysis of ovarian gene expression during ovulation using differential display. *Methods in Molecular Biology*.

[29] Murakami M., Taketomi Y., Miki Y., Sato H., Hirabayashi T., Yamamoto K. (2011). Recent progress in phospholipase A2 research: from cells to animals to humans. *Progress in Lipid Research*.

[30] Kurusu S., Iwao M., Kawaminami M., Hashimoto I. (1998). Involvement of cytosolic phospholipase A2 in the ovulatory process in gonadotropin-primed immature rats. *Prostaglandins Leukotrienes and Essential Fatty Acids*.

[31] Kurusu S., Sapirstein A., Bonventre J. V. (2012). Group IVA phospholipase A2 optimizes ovulation and fertilization in rodents through induction of and metabolic coupling with prostaglandin endoperoxide synthase 2. *FASEB Journal*.

[32] Fujishima H., Sanchez Mejia R. O., Bingham C. O. (1999). Cytosolic phospholipase A_2_ is essential for both the immediate and the delayed phases of eicosanoid generation in mouse bone marrow-derived mast cells. *Proceedings of the National Academy of Sciences of the United States of America*.

[33] Funk C. D., Song W. C., FitzGerald G. A. (2009). Prostaglandins and other lipid mediators in reproductive medicine. *Yen & Jaffe's Reproductive Endocrinology*.

[34] Takahashi T., Morrow J. D., Wang H., Dey S. K. (2006). Cyclooxygenase-2-derived prostaglandin E2 directs oocyte maturation by differentially influencing multiple signaling pathways. *Journal of Biological Chemistry*.

[35] Murakami M., Kudo I. (2003). Cellular arachidonate-releasing functions of various phospholipase A2s. *Advances in Experimental Medicine and Biology*.

[36] Richards J. S., Russell D. L., Ochsner S., Espey L. L. (2002). Ovulation: new dimensions and new regulators of the inflammatory-like response. *Annual Review of Physiology*.

[37] Gilroy D. W., Newson J., Sawmynaden P., Willoughby D. A., Croxtall J. D. (2004). A novel role for A2 isoforms in the checkpoint control of acute inflammation. *The FASEB Journal*.

[38] Milne S. A., Henderson T. A., Kelly R. W., Saunders P. T., Baird D. T., Critchley H. O. D. (2005). Leukocyte populations and steroid receptor expression in human first-trimester decidua; regulation by antiprogestin and prostaglandin E analog. *The Journal of Clinical Endocrinology & Metabolism*.

[39] Ristimäki A., Sivula A., Lundin J. (2002). Prognostic significance of elevated cyclooxygenase-2 expression in breast cancer. *Cancer Research*.

[40] Yao N., Yang B. Q., Liu Y. (2010). Follicle-stimulating hormone regulation of microRNA expression on progesterone production in cultured rat granulosa cells. *Endocrine*.

[41] Iwaya T., Yokobori T., Nishida N. (2012). Downregulation of miR-144 is associated with colorectal cancer progression via activation of mTOR signaling pathway. *Carcinogenesis*.

[42] Sobinoff A. P., Pye V., Nixon B., Roman S. D., McLaughlin E. A. (2010). Adding insult to injury: effects of xenobiotic-induced preantral ovotoxicity on ovarian development and oocyte fusibility. *Toxicological Sciences*.

